# Experiences with a structured conversation tool: a qualitative study on feasibility in general practice in Norway

**DOI:** 10.1080/02813432.2022.2076396

**Published:** 2022-05-19

**Authors:** Cathrine Abrahamsen, Morten Lindbaek, Erik L. Werner

**Affiliations:** a MD, PhD student, Department of General Practice, University of Oslo, Oslo, Norway; b MD, PhD, Senior Researcher, and Professor of General Practice, Department of General Practice, University of Oslo, Oslo, Norway

**Keywords:** General practitioner (GP), conversation tool, cognitive therapy, work-focused cognitive therapy, medically unexplained physical symptoms (MUPS), sick leave

## Abstract

**Objective:**

To study the feasibility of a structured conversation tool (ICIT) in Norwegian general practice.

**Design and participants:**

A structured conversation tool with elements from Cognitive Behavioral Therapy (CBT) was developed for use at the encounter in general practice to increase patient’s self-coping ability and the GPs management and sick leave assessment in patients with MUPS. Eight GPs received training and used the ICIT on 49 patients with MUPS. The physicians were gathered into two focus groups. The interviews were recorded on tape, transcribed, and analyzed with systematic text condensation.

**Main outcome measures:**

The aim of our study was to examine any benefit and the feasibility of the ICIT in general practice.

**Results:**

The physicians found the ICIT helpful to sort out, clarify and concretize the patients' issues. They felt less fatigued as patients took on a greater responsibility for their own recovery and reported a greater satisfaction and better management with the patients. A salutogenic approach gave the physicians greater insight into their patients’ lives and their issues, opening for new treatment options and aiding in recovery. By focusing on the patient’s potential capabilities despite their medical condition, some physicians experienced that patients on sick leave returned to work quicker.

**Conclusions:**

The GPs in this study reported that the ICIT was helpful in consultations with patients due to unspecific medical conditions and facilitated a sense of competence for the physician.

**KEY POINTS**
GPs need communication skill training for integrated treatment and sick leave assessment for patients with Medically Unexplained Physical Symptoms (MUPS).
•The GPs experienced that the structured conversation tool was beneficial in structuring, clarifying, and substantiating the patient's problems.
•The GPs experienced a greater insight into their patients and their issues, opening new treatment options and aiding in recovery.
•The GPs experienced patients’ quicker recovery and returns to work by starting immediate treatment using the conversation tool.

## Introduction

Medically unexplained physical symptoms (MUPS) are a group of symptoms and ailments without objective findings [1]. Patients with MUPS are common in general practice [2] and are associated with reduced quality of life, high use of health services and costs associated with lost productivity [3–5]. Patients with MUPS are often dissatisfied with their medical treatment and often feel stigmatized and not taken seriously [6]. Many GPs experience frustration in meeting patients with MUPS in the absence of precise diagnostics and well-defined treatment options [7].

Studies show that the doctor's work with sick leave is particularly demanding when the patient does not have a clear diagnosis [8,9]. Sick leave may contribute to avoidance behavior [10] with negative consequences for the individual in work situations, even for those returning to work full time [11].

Research suggests that GPs need communication skill training with a special emphasis on difficult situations in sick-listing practice [8]. A systematic review of the management of MUPS in primary care suggests exploring a feasible approach for integrated treatment in routine primary care [12].

Salutogenesis is focusing on what contributes to good health rather than what creates health problems [13]. GPs do not often communicate with their patients about coping, and communication skills training for GPs in this field is suggested [14].

The finding of an increased sense of competence in learning cognitive conversational techniques is congruent with several studies [15,16]. In clinical practice, CA recognized a need for a structural conversational tool that could both increase patients’ self-coping ability and the doctor's management and sick leave assessment in patients with MUPS. A conversation tool, the “Individual Challenge Inventory Tool” (ICIT), was therefore developed in order to meet this experienced need which also is suggested in the literature [8,12].

The psychologist Albert Bandura defines self-efficacy as people's belief in their capabilities to exercise control over their own functioning. According to Bandura's social learning theory, self-efficacy can be learned through observational learning [17].

Bandura proposed that this type of learning involved four different stages: attention, retention, reproduction, and motivation. Consequently, we tailored our training program according to these four different stages in an attempt to increase the GPs self-efficacy.

The aim of our study was to examine any benefit and the feasibility of the ICIT in general practice. We defined the feasibility of ICIT whether ICIT could be integrated spontaneously into daily care in general practice with no pre-planning and whether the physicians found it beneficial particularly in their management of patients with MUPS.

## Design, material, and methods

We have conducted a qualitative study based on two focus group interviews of GPs in Vestfold and Telemark County, Norway. In general, Norwegian general practice differs from most other countries by having longer durations for consultations. This allows GPs to include conversation strategies like Cognitive Behavioral Therapy (CBT) in their practice, which in other countries would be regarded as a tool for specialist care.

## Data collection

**Figure 1.**
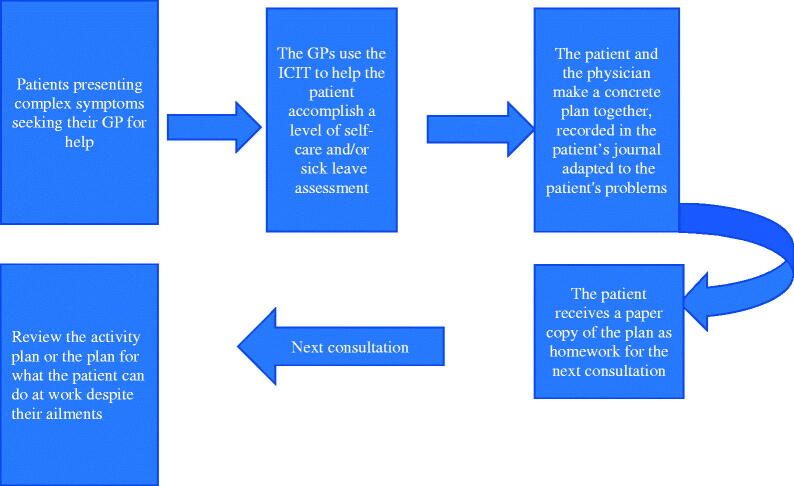
The use of ICIT.

By using the ICIT, the patient is first encouraged to define his or her current problems, then to sort and prioritize them. The problems may be different health issues, or consequences resulting from these. Thereafter, the patient is challenged to make a plan including some concrete measures aimed at solving one or more of their health issues. If any of these issues are work-related, the patient is also motivated to explore which possible scenarios within their current work situation could be modified to make it possible to stay at work. The overall goal is to make the patients themselves be proactive in their own treatment. The patient and the physician make a concrete plan together, recorded in the patient’s journal. Homework is a crucial part of CBT; the patient receives a copy of the plan and the agreed actions to do for the next consultation (Figure 1).

The ICIT was initially evaluated through a pilot study involving 7 general practitioners in specialization in 2019. The participants tested the conversation tool on their own patients for 4 weeks and it was revised based on their feedback. We invited eight GPs to participate in our study. All GPs were participating in a 2-year compulsory supervisory group as part of their specialization in general medicine, led by CA. The physicians had no prior knowledge of CBT, and they all gave oral and written consent to participate in the study.

The participants were trained in the use of the ICIT for a total of 18 h over 4 days in an 8-week period. According to Bandura's social learning theory, we tailored the training of the GPs into four steps: (i) attention by observing the practical use of the ICIT through two videos, (ii) theoretical training to retain and internalize what they had seen, (iii) reproduce the behavior into action through role-play example scenarios from their own practice which they had experienced as particularly challenging. The GPs video-recorded a consultation using the ICIT with the patient's written consent and then reviewed the videos with each other's guidance. The fourth element of Bandura’s learning theory is (iv) to motivate the GPs to imitate the behavior they have observed.

The participants then adopted the ICIT on their own patients in practice. All the patients were orally informed about the study and the GPs emphasized to the patients that the aim of the study was the physician’s experience, not the patient’s.

Following this period of practical use, the GPs were invited to a focus group interview. In total, seven male and one female physician (age 31–47 years) in general practice were divided into two focus groups for interviews. The interviews were conducted by CA with ELW as an observer.

## Analysis

The interviews were recorded on tape, transcribed, and analyzed with systematic text condensation [18]. Manual analysis was performed with color codes without the use of computer programs.

The analysis involved the following steps:

(i) review of both group interviews by all three authors individually, to gain full oversight of data/material to then identify overall topics (themes).
The various topics were then discussed in several meetings between the authors, in order to (ii) develop code groups from the preliminary themes, identify and code meaning units reflecting the GPs experiences of using the ICIT on their patients, (iii) establish subgroups exemplifying the vital aspects of each code group, condense the contents of each sub-group and identify illustrating quotes, and (iv) condense and reformulate the content of each code group from the text to more general statements. The themes arose from the data and were not predetermined. The analysis of results was summarized into four main themes.

The participants were not given the opportunity to comment on the choice of a topic or the result of the analysis.

## Results

The GPs used the ICIT in their own practice in a minimum of 4 patients each, of a total of 49 patients with MUPS. Based on the text analysis, the GPs experiences can be summarized into four themes: 1) physicians' experiences with the ICIT as a structured work methodology, 2) the doctor`s perception of the patient's experience with the use of the ICIT, 3) the ICIT’s scope of application for the assessment of sick leave and treating patients diagnosed with MUPS, and 4) shortcomings and challenges in utilizing the ICIT in practice.

### The physicians found the ICIT helpful to sort out, clarify and concretize the patients' issues

All participating GPs reported that the ICIT was beneficial in structuring, clarifying, and substantiating the patient's problems. By eliciting a self-formulation of the problems from the patient's perspective, the physicians found that the ICIT was time-saving and provided a more rapid and comprehensive understanding of the patient. One physician told the story of a patient who had stopped driving due to anxiety and had used public transport instead to travel to work.

The patient was then concerned about contracting covid-19 on the bus and this contributed to her demand for sick leave:

“I felt that this was good because we were able to isolate a specific problem. I don't think she thought about it too much because in fact she would have found it so much easier if she had driven a car. And that was the main reason for her problems” (Participant number 5).

The treatment thus became an exposure plan directed at the patient starting to drive again.

By using the ICIT, the physicians were less tired in their work with this challenging group of patients. The patients were given more responsibility with specific, written homework which meant treatment was ongoing and could continue between consultations. Descriptions such as "getting past the symptoms", "not just nonsense", "a lighter feeling inside me" and "but here they actually have to work with the problem" were used.

“I think some people just come for approval and acceptance, and it gets tiring for us. Whereas we can bring this up and it can be used to make a plan for them to improve their personal situation. It helps us in helping them.”

(Participant number 8)

By asking patients the question "Is there anything that used to please you that you don’t have the energy for anymore?” it became clearer to the physicians what the actual consequences of the patient’s problems were in their daily lives. In that respect, the physicians saw the patients in a new light, and this resulted in new treatment options in the form of an activity plan based on the patient's pre-symptomatic lifestyle and revisiting activities that they previously derived pleasure from.

All participating physicians felt that adopting the ICIT as a work tool gave them encouragement and a sense of enhanced competence as therapists. They fed back terms such as: "It was useful for my self-esteem as a doctor" and "A useful tool to employ while patients wait for further treatment by specialists".

One physician talked about a patient who recovered before consultation with a psychologist:

“What I often do is to refer my patient to a psychologist, and then my part in the treatment is done in a way… but now we can do something to improve the situation in a primary care setting, while waiting for specialist treatment. In one case, it turned out to be the correct way forward and treating this lady using ICIT was enough. She did not need to see a psychologist in the end, as consulting me was enough.”

(Participant number 1)

An interesting observation we did, was that GPs who reported poor communication skills ahead of the study displayed the most enthusiasm for using the ICIT in practice.

### The doctor's perception of the patient's experience with the use of the ICIT

The physicians experienced that the patients achieved a sense of control by following an activity plan divided up into small steps. In this way, some patients learned an important self-help tool. Previously, in patients with MUPS, some of the GPs expressed frustration by not having any specific treatment to offer and often got the impression that the patients were dissatisfied with their treatment. The ICIT seemed to provide these doctors, particularly the tool they needed to be able to offer the patients something specific, like their activity plans.

“At the subsequent consultation a patient told me about both good and bad experiences since the last time, but overall, she felt a sense of achievement as she fulfilled the agreed actions from the activity plan.”

(Participant number 8)

Based on how the doctor perceived the patients' experiences the physicians reported more grateful patients. Some patients also felt better having been helped by their GP rather than by a psychologist.

“When the patient came back to me, they told me that they would rather have me to talk to and have as a psychologist. And primarily, it was the ICIT that we had done.”

(Participant number 2)

Several of the physicians recounted patient experiences where the patient discovered the consequences that their personal challenges had in their lives, such as how social withdrawal had increased depressive symptoms and loneliness.

“When we employed the ICIT methodology, we found that the patient became more engaged and looked more positively upon life. She found that she could do something helpful to help herself." (Participant number 8)

### The ICIT’s scope of application

In assessing sick leave, the physician could ask the patient: “What things at my workplace would actually be good for me, despite how I am feeling right now?”, a question from the ICIT, encourages the patient to reflect positively on their situation.

The patients reflected on what they could do at work, instead of focusing on their limitations and what they cannot do. The participating physicians felt they were no longer merely there as a provider of sick leave. When the patients were able to master the small things in their everyday life, they gradually gained the energy and enthusiasm to want to return to work.

One physician expressed:

“Many patients just accept they are on sick leave and are passive. But by using the ICIT they get homework and must work and put some effort into helping to improve their own health. Sick leave is not a holiday. "

(Participant number 3).

Another physician described how she used the methodology to act out a difficult situation at work, together with the patient.

“By using the ICIT it definitely shortened the patient’s period of sick leave. Previously, I would have simply referred the patient to a psychologist, that may have left the patient not actively engaged in trying to improve her health whilst waiting for another appointment. I have the feeling that it would not have simply ended with 3 to 4 weeks sick leave….“

(Participant number 1)

The participating physicians used the ICIT on patients with MUPS. They referred to both this group of patients and individuals who they experienced as particularly demanding and challenging. The ICIT contributed to better contact with the patient and a more positive atmosphere in consultations.

One physician expressed:

“Some patients still come to see me with all their symptoms despite their medical treatment being fulfilled. Before, I did not know how to handle these patients. But now I can use the ICIT to help them.” (Participant number 8)

### Limitations and challenges with utilizing the ICIT

The GPs recognized the importance of creating a realistic activity plan together with the patient so that the patient felt it was achievable. One patient was afraid the physician would be disappointed if the entire activity plan was not accomplished.

When introducing the ICIT at the beginning of the consultation, it was important to inform the patient about the concept of the ICIT and the methodology behind it. Some patients were reluctant to disclose all their issues at one time.

“One patient was very talkative, and we dug so deep that we ended up with too many problems. There was a lot of talk about each individual topic which was a bit problematic, but then you must just be professional and cut to the chase.” (Participant number 5)

If the patient did not go home with a written activity plan, the tasks the GPs and patient had discussed would not be accomplished. Another physician experienced that one patient got angry and he felt that the ICIT trivialized his challenges:

“Then he completely lost it, he got angry and told me that his problem could not be simply solved by going for a walk or meeting a friend… That was a bit uncomfortable” (Participant number 6)

## Discussion

In all kinds of treatment, establishing good contact with the patient is crucial. Physicians need tolerance and understanding in treating patients with MUPS. According to the GPs in our study, they experienced an increased sense of competence in adopting the ICIT and felt less tired. All the physicians found it useful to refine and concretize the patient's challenges, as well as to some extent reflect the responsibility for improvement back to the patient themselves.

## Methodological considerations

The development of the ICIT is an innovative work tailored for the primary care setting. CBT is established as an effective treatment for anxiety and depression [19]. The ICIT is composed of different cognitive working tools such as Socratic questions, activity plans and homework to enhance its validity.

The participating physicians were all recently educated GPs and therefore did not know their patients very well. This may have made it easier to implement something new in the consultations. For physicians with long experience and a more established consultation style, change can be more difficult [15]

Despite the number of participants is limited, the information power (IP) from the data was deemed as being sufficient [20]. Factors pertaining to high IP in this study included a narrow study aim, purposive and specific sampling, and a strong interview dialogue. The physicians that participated had experience from 49 patients’ consultations, which is regarded as sufficient to assess the feasibility of the ICIT.

CA, who conducted the interviews, is a GP and has comparable experiences with using the ICIT in consultations on patients with MUPS. This position made her familiar with the participants’ experiences, making it easier for her to probe deeper for detailed information. On the other hand, her being the author of the ICIT and the participants' supervisor in the supervision group were also significant elements of her preconceptions which could influence the dialogue and analysis.

This may have also affected the participants' feedback and interest, which is why we focused on an open conversation in both group interviews. The importance of revealing both good and bad experiences was emphasized at the introduction of both group interviews.

We were especially interested in participants sharing experiences. In this way a wide variety of experiences surfaced. In group interviews, social norms within the group can become governing and thus prevent disagreements from being expressed [21]. In our study, all the GPs knew each other well, which provided a trusting climate for learning and exercises with the ICIT and the focus group interview. We chose CA to conduct the group interviews due to her knowledge of the participants and the tool in question. The participants were divided into two groups to give each one the participants enough time to talk.

A methodological strength is that we have based the study on open group interviews with the physicians’ experiences, where the topics emerged from the physicians' associations with each other. Besides that, the categorization of themes and the interpretation of the material were done individually, and then discussed and compared by the authors in several meetings.

This study focuses on the participants’ experiences with the ICIT. Any report on experiences at the patient level is therefore a methodological challenge because it necessarily was the physician's interpretation of the patients’ experiences with the ICIT that was reported. In this study, we have primarily carried out a cross-cutting, thematic descriptive analysis of a manifest content to describe the doctors' experience of the intervention ICIT. The GPs interpretations were based on both the patients’ verbal and non-verbal feedback, as well as their compliance.

The aim of our study was to assess the feasibility of the ICIT. We emphasized that the physicians should not have prior knowledge or special interest in CBT, nor use pre-planned sessions for the use of ICIT, in order to increase the validity in a real primary care setting. The participating GPs fitted this criterion. The ICIT may be most useful among GPs with an interest in the patient's life and in helping patients with MUPS. On the other hand, the GPs ability to create contact with patients can be learned. GPs who devalue their psychological skills are less likely to participate in training [22]. The lack of interest in such training may also be due to a lack of competence. Possibly, a tool like ICIT could be found useful also for GPs with minor interest and competence in CBT and hereby increase their communication skills. The observation we made of those GPs in our groups who increased their enthusiasm for the ICIT tool may be interpreted as increased skills. This may suggest that the ICIT is feasible in general practice. CBT is learned most effectively using modeling and role-playing, in addition to theoretical training (24). This corresponds to the training of the GPs in our study and should therefore be considered a strength.

## What is known from before – what does this study add?

The finding of an increased sense of competence in learning cognitive conversational techniques coincides with other studies [15,16]. We compared our findings with those published by Patel et al. [16] where 50 GPs tested the feasibility of a shorter training in 90 min on how to utilize cognitive behavioral skills within their consultation on patients with MUPS. The GPs participating in this study increased their confidence in their management of the patients.

In our study, the GPs were trained for 18 h, and we conducted role-play exercises among the participants in addition to the demonstration on video, while the Patel study only demonstrated it on video. Thirdly, the participants in our study filmed a patient consultation with one of their own patients while using the ICIT. It was observed that the GPs used the ICIT mostly in the same way. Finally, our participants used the ICIT at the encounter while those in the Patel study were trained to use the methodology without any specific tool.

The combination of theoretical and practical training, in addition to providing the ICIT as a written conversation tool, might have been important for our results. Our finding of the GPs experiences a quicker recovery and return to work by starting immediate treatment using the ICIT is in accordance with other studies [23,24]. Work-focused cognitive therapy contributes to faster recovery compared to traditional cognitive therapy [25]. When the GPs use the ICIT, the patient is challenged to reflect on what they can do at work, despite their health problems, and in this way related to work-focused cognitive therapy.

## Implications

This study suggests that the ICIT provides increased insight into the patients with MUPS’s health complaints which in particular may be helpful in the assessment of the patient’s work abilities. Our findings indicate how the ICIT can help to structure the consultation GPs have with patients with MUPS. The GPs experienced an increased sense of management in their meetings with a challenging group of patients and felt less tired. This may increase the GPs endurance, and perhaps prevent the danger of overworking and burnout. According to the participants in our study and our feasible criteria, the ICIT was deemed easily feasible in Norwegian general practice. We suggest further studies to show any implications at the patient’s level.
